# Human Pythiosis, Brazil

**DOI:** 10.3201/eid1105.040943

**Published:** 2005-05

**Authors:** Sandra de Moraes Gimenes Bosco, Eduardo Bagagli, João Pessoa Araújo, João Manuel Grisi Candeias, Marcello Fabiano de Franco, Mariangela Esther Alencar Marques, Leonel Mendoza, Rosangela Pires de Camargo, Silvio Alencar Marques

**Affiliations:** * Universidade Estadual Paulista, Botucatu, São Paulo, Brazil;; † Universidade Federal de São Paulo, São Paulo, Brazil;; ‡ Michigan State University, East Lansing, Michigan, USA

**Keywords:** Epidemiology, Latin America, Pythium, subcutaneous, rDNA ITS, molecular sequencing data

## Abstract

Pythiosis, caused by *Pythium insidiosum*, occurs in humans and animals and is acquired from aquatic environments that harbor the emerging pathogen. Diagnosis is difficult because clinical and histopathologic features are not pathognomonic. We report the first human case of pythiosis from Brazil, diagnosed by using culture and rDNA sequencing.

Pythiosis is a cutaneous-subcutaneous disease of human and animals that occurs primarily in tropical and subtropical areas of the world. It is caused by the aquatic funguslike organism *Pythium insidiosum* (kingdom Straminipila, phylum Oomycota, class Oomycetes) (1). Although pythiosis is not caused by a true fungus, the pathogen has some morphologic characteristics in common with fungal members of the order Zygomycetes, mainly in histopathologic sections. This disease is a common cutaneous and intestinal disorder in horses, cattle, dogs, and cats (2,3). Human pythiosis may appear in a cutaneous-subcutaneous form with lesions on the limbs, periorbital and facial areas, and corneal ulcers. Pythiosis can also be a systemic disease involving the vascular system, which usually causes arterial occlusion. The systemic form has been documented in numerous patients, most from Thailand, with thalassemia. The remaining cases have been from Australia (2 patients), United States (2 patients), and 1 each from Haiti, Malaysia, and New Zealand (4,5).

*Pythium insidiosum* can be cultured on Sabouraud dextrose or brain heart infusion agar at 37°C from clinical material, such as pus, lesion exudates, and biopsy material. The characteristic asexual biflagellate zoospores, important for the diagnosis, can be induced in liquid media but not in solid cultures. In tissues, *P. insidiosum* stains well with Gomori methenamine silver and periodic acid-Schiff stain. The organism appears with broad, branched, and sparsely septate or nonseptate hyphae, often identified as fungal elements of the zygomycetes (6). Conventional diagnosis is based mainly on immunofluorecence and immunoperoxidase procedures, which have proved specific in tissues of persons, cats and dogs with pythiosis. Serologic tests, such as enzyme-linked immunosorbent assay (ELISA) and immunodiffusion, have been also used to diagnose pythiosis (7). Molecular diagnostic assays, such as nested polymerase chain reaction and a species-specific DNA probe from the ribosomal DNA complex, have been useful to identify *P. insidiosum* in the absence of culture (8,9). This article reports the first case of human pythiosis in continental Latin America in a patient from Brazil, and diagnosis was confirmed by molecular taxonomy.

## The Study

**Figure 1:**
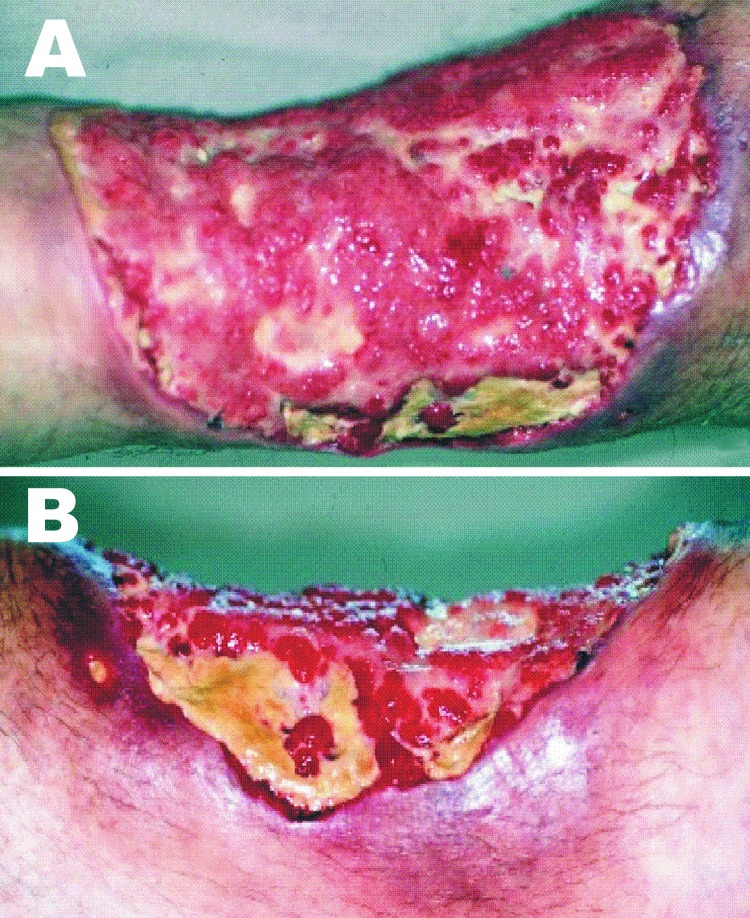
Clinical aspects of the lesion when the patient sought treatment at the University Hospital. The figure depicts the wide extension of the lesion in a frontal (A) and in depth (B) medial views.

**Figure 2:**
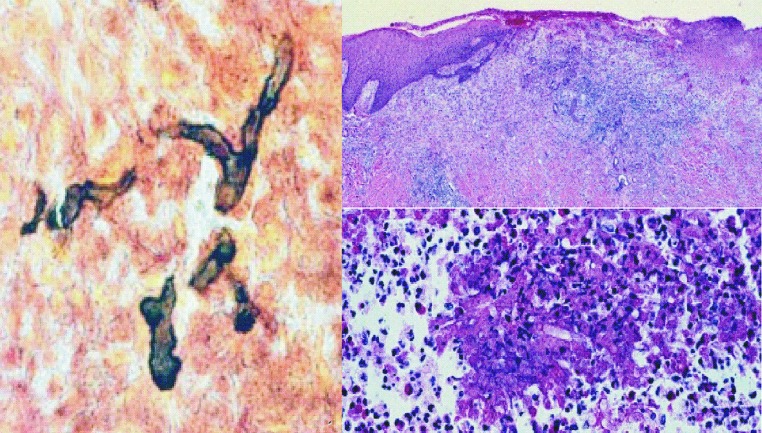
Biopsied section of the lesion: A) branch and broad hyphal fragment of Pythium insidiosum (Gomori methemine silver, magnification ×200); B) hyphal fragment surrounded by inflammatory cells (hematoxylin and eosin [HE], magnification ×200); C) hyphal fragment surrounded by eosinophilic material, which suggests a Splendore-Hoeppli phenomenon (HE, magnification ×200).

A 49-year-old policeman was admitted on May 2002 to the dermatology division of the university hospital for the treatment of a skin lesion on his leg, initially diagnosed as cutaneous zygomycosis. The patient stated that a small pustule developed on his left leg 3 months earlier, 1 week after he fished in a lake with standing water. The pustule was initially diagnosed as bacterial cellulitis; it was treated with intravenous antimicrobial agents with no improvement. A biopsy of the lesion showed a suppurative granulomatous inflammation associated with several nonseptated hyphae (shown by Gomori methenamine silver stain), a finding that led to the diagnosis of zygomycosis. The treatment was then changed to amphotericin B. After receiving 575 mg of accumulated dosage plus 2 surgical debridements of the lesion, the patient showed only slight improvement; he was then referred to our university hospital. At admission, the physical examination showed a tibial ulcer 15 cm in diameter with an infiltrating and nodular proximal border (Figure 1). Serologic testing showed the following laboratory values: leukocyte count 4,200/mm^3^ with 9% eosinophils, glucose 100 mg/dL, and negative serologic test results for HIV infection. Azotemia, hypokalemia, and normocytic anemia were observed as adverse effects of the previous amphotericin B treatment. Results of a second biopsy from the border of the ulcer again suggested zygomycosis (Figure 2).

Oral itraconazole, 400 mg/day, was prescribed. Although the initial response was encouraging, at the end of the first month of itraconazole administration, the lesions recurred. Potassium iodine, 4 g/day, was also prescribed, but no clinical improvement was detected after 2 months. Attempts to isolate the agent in the hospital laboratory yielded negative results. With the progression of the disease, an extensive surgical debridement was considered and a computed tomographic scan defined the limits of the infection. A course of amphotericin B was begun 1 week before the intervention, which included total removal of the fascia lata. Amphotericin B was maintained until discharge from the hospital on day 103. A late skin graft produced an acceptable recovery. The excised tissue supplied enough material for culture and molecular assays in our laboratory, which led to a conclusive diagnosis.

The excised tissue was immediately washed in 70% alcohol and sterile saline, minced in small fragments (2 × 2 mm) and cultured on Sabouraud dextrose and potato dextrose agar (PDA, Oxoid Ltd., Basingstoke, Hampshire, UK), plus chloramphenicol and gentamicin (50 μg/ml), at 25°C. After the tissues underwent a period of growth, slide cultures were carried out with PDA medium.

The DNA extraction was performed according to the glass beads protocol proposed by van Burik et al (10). Polymerase chain reactions (PCRs) were carried out in 25 μL of reaction mixtures buffered with 20 mmol/L Tris-HCl (pH 8.4), containing 10 ng of genomic DNA, 20 pmol/L of each primer, 1.5 mM MgCl_2_, 50 mmol/L KCl, 0.2 mmol/L of each deoxynucleoside triphosphate, and 0.2 U of Taq DNA polymerase (Amersham Biosciences Corp, Piscataway, NJ, USA). PCRs for elongation of rDNA-ITS region were performed in a thermocycler (MJ Reasearch, Inc, Waltham, MA, USA), with an initial cycle at 94°C for 5 min, followed by 25 cycles at 94°C (1 min), 60°C (2 min), and 72°C (2 min) and a final extension of 7 min at 72°C. The ITS regions were amplified by using the universal ITS4 (5´–TCC TCC GCT TAT TGA TAT GC–3´) and ITS5 (5´–GGA AGT AAA AGT CGT AAC AAG G–3´) set of primers. The amplicons were visualized in ethidium bromide-stained 1% agarose gel and purified in GFX column (GFX PCR DNA and Gel Band Purification Kit, Amersham Biosciences).

Both strands were sequenced in an ABI Prism model 377 DNA Sequencer (Applied Biosystems, Foster City, CA, USA). The sequencing reactions were performed with Big Dye Terminator v3.1 Cycle Sequencing Kit (Applied Biosystems), 1.6 pmol/L of each ITS4 or ITS5 primer and 60 ng of purified DNA. Ultra pure water was used to complete a volume of 20 μL. The elongation of the ITS region was performed in a thermocycler (Mastercycler gradient, Eppendorf, Hamburg, Germany) with 40 cycles at 96°C (10 s), 50°C (5 s), and 60°C (4 min). The ClustalW program was used to align nucleotide sequences. The obtained sequences were submitted for analysis to GenBank by using BLAST (http://www.ncbi.nlm.nih.gov/BLAST).

**Figure 3:**
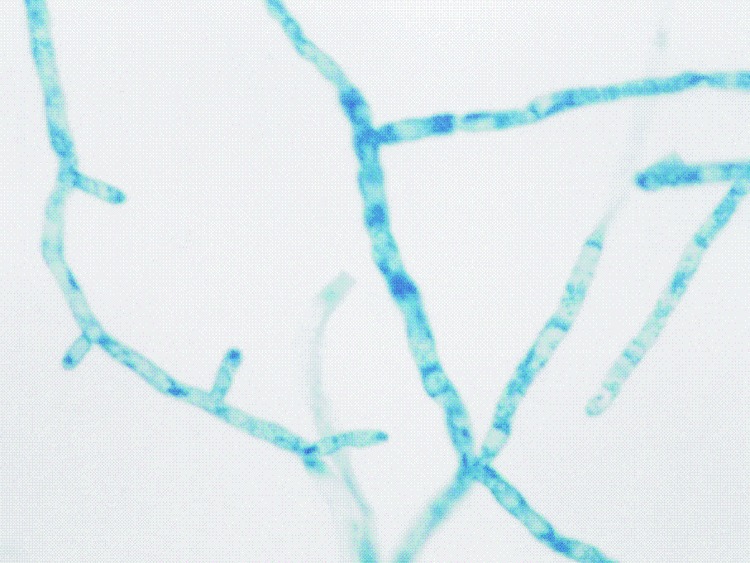
Slide culture illustrating the broad, branched, and sparsely septate hyphae of Pythium insidiosum (lactophenol cotton-blue, magnification ×40).

Pure, colorless, membranous colonies grew on Sabouraud and PDA from almost all samples. On microscopic examination, slide cultures stained with lactophenol cotton-blue showed broad, branched, and sparsely septated hyphae, without fruiting bodies (Figure 3), which later were identified as colonies of *P. insidiosum*.

**Figure 4:**
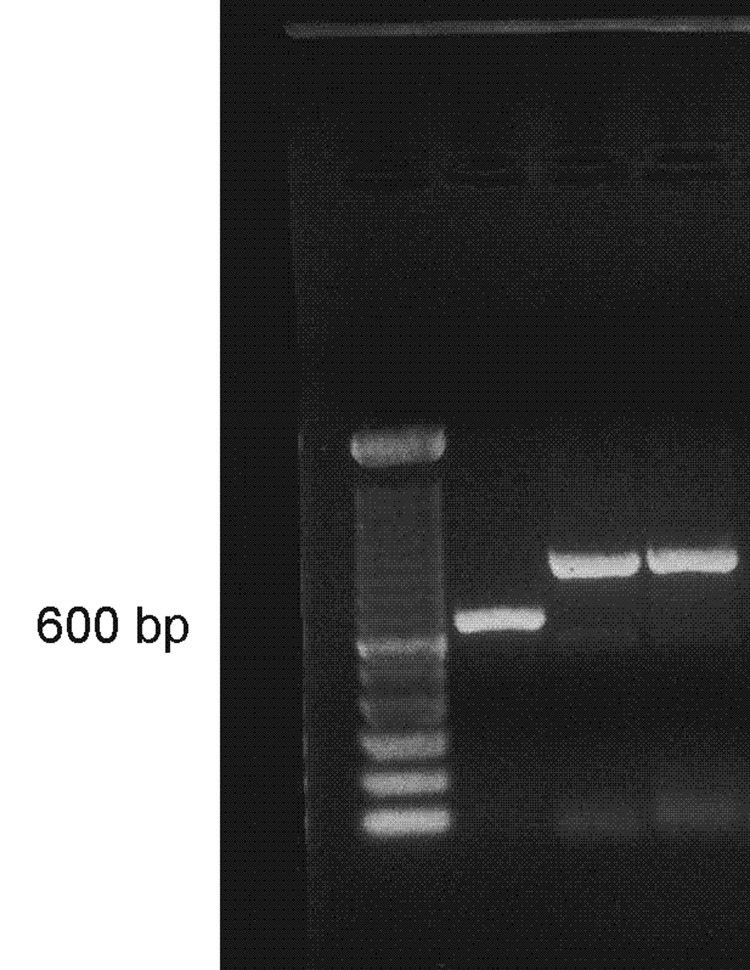
Polymerase chain reaction with ITS4/ITS5 primers: lane 1, ladder 100 bp; lane 2, positive control showing Pythium brasiliensis band at position 634 bp; lanes 3 and 4, P. insidiosum isolate B01 showing band at position ≈850 bp.

The first round of amplification with the generic primers for fungi (ITS4/ITS5) produced weak bands; in a second round, double PCR, defined and sharp bands of approximately 850 bp were seen (Figure 4). After being purified and sequenced in both directions, bands produced chromatograms with a resolved sequence of 593 bases. After BLAST analysis, the sequence showed 100% of identity with the deposited sequences of *P. insidiosum* (accession nos: AY151166, AY151165, AY151159), Schurko, et al. (11), with full annealing from base numbers 253 to 845, which include the almost complete gene 5.8S and the complete variable ITS2 region.

## Conclusions

In the present case, a diagnosis of zygomycosis was initially suggested by the histopathologic examination carried out in 2 different laboratories. The patient had no evidence of immunologic disorders and no clinical manifestation of thalassemia. The infection was more likely acquired during water-associated leisure activities, the most common source of the infection among patients with pythiosis (12). Several antifungal drugs were used, but the lesions did not improve. On the contrary, during the 14 weeks of chemotherapy the status of the lesion worsened, and amputation of the patient's leg was considered. After extensive surgical debridement to remove the lesion, however, a cure was achieved. Although all mycologic features and epidemiologic data indicated possible infection by *P. insidiosum*, only after sequencing rDNA ITS was the diagnosis of pythiosis definitively established. We believe this is the first case of human pythiosis described in South America, although similar cases might have been misdiagnosed as putative cases of zygomycosis in humans, as others have noted (6).

Cases of subcutaneous pythiosis with dissemination to internal organs have been described in equines in Brazil (13). Subcutaneous pythiosis in calves and equines seems to be common in the central and southeast regions of Brazil. More recently, an outbreak of cutaneous pythiosis was reported in 2 herds of crossbred wool sheep in the northeast region (14). This pathogen likely occurs in different regions of Brazil because of a prevalent tropical climate and abundant sources of water.

The fragment of rDNA sequenced in the present case included the complete variable ITS2 region and almost the complete coding sequence of 5.8S. This fragment showed 100% identity with the isolates M16, M12, and 339 that characteristically belong to the cluster I of the American isolates (11). The M16 isolate was obtained from a corneal lesion of a Haitian patient and the others were from Costa Rican equines. A strain of *P. insidiosum* from a Brazilian equine (394) located in the same American cluster showed a similarity of 99% with our *P. insidiosum* isolate.

Direct DNA sequencing, mainly of rDNA regions, proved to be an important and consistent tool for the taxonomic identification, of different groups of organisms (9). In this case, a molecular approach was decisive in identifying and diagnosing this life-threatening disease. Our experience indicates that other cases of pythiosis may have been misdiagnosed as cases of cutaneous zygomycosis. Although zygomycosis has a widespread distribution and is well known to physicians, this familiarity is not the case for human pythiosis. Health professionals, therefore, should be aware of the importance of an accurate diagnosis of this condition and know how to differentiate it from zygomycosis, since pythiosis has a completely different prognosis and requires different therapy.

We thank the State of São Paulo Research Foundation (FAPESP) for the financial support.
